# Editorial: BCR Signaling and B Cell Activation

**DOI:** 10.3389/fimmu.2020.00045

**Published:** 2020-01-28

**Authors:** Wanli Liu, Pavel Tolar, Wenxia Song, Tae Jin Kim

**Affiliations:** ^1^MOE Key Laboratory of Protein Sciences, Center for Life Sciences, Collaborative Innovation Center for Diagnosis and Treatment of Infectious Diseases, School of Life Sciences, Beijing Key Lab for Immunological Research on Chronic Diseases, Institute for Immunology, Tsinghua University, Beijing, China; ^2^Laboratory of Immune Receptor Activation, Francis Crick Institute, London, United Kingdom; ^3^Department of Cell Biology and Molecular Genetics, University of Maryland, College Park, MD, United States; ^4^Department of Immunology, Sungkyunkwan University School of Medicine, Suwon, South Korea

**Keywords:** B cell, B cell receptor (BCR), B cell activation, antibody, antigen, imaging, co-receptor, cytoskeleton

Signaling via the B cell receptor (BCR) is essential for B cell survival and development, and antibody production in both physiological and pathological conditions. The nature of BCR signaling is varied in different subpopulations and developmental stages of B cells and can be classified into tonic, chronic active, and priming signaling (1, 2). Whereas, tonic BCR signaling is required for B cell survival and development, chronic active BCR signaling supports the continuous proliferation of B cell lymphoma cells, and antigen-driven priming signaling is important for the initiation of B cell activation and differentiation into antibody-secreting cells. Although the detailed molecular mechanism underlying diverse BCR signaling patterns has been an elusive question in immunology, our knowledge on this regard has been significantly expanded in recent years due to the development of advanced imaging and next-generation sequencing technologies. The aim of this Research Topic, including seven original research articles and five review articles, is to highlight the current understanding of BCR signaling and its relationship with health and disease.

The BCR-mediated signaling in homeostatic conditions depends on the availability of antigens and is delicately regulated by co-stimulatory and co-inhibitory receptors. Co-inhibitory receptors limit BCR signaling in order to prevent B cells from hyperactivation and maintain B cell homeostasis. Among many inhibitory receptors, the proteins of the Sialic acid binding immunoglobulin-like lectin (Siglec) family play important roles in regulating BCR signaling. Meyer et al. reviews Siglecs in B cells, highlighting the interplays between CD45 and CD22 (Siglec-2), CD22 and Galectin-9, and their influence on BCR signaling. Both CD22 and Siglec-G contain immune receptor tyrosine-based inhibitory motifs (ITIMs) within their cytoplasmic tails and recruit the tyrosine phosphatase SHP1, which inhibits B cell signaling. Whereas, CD22 mainly functions in conventional B cells, Siglec-G inhibit the constitutive BCR signaling in autoreactive B-1a cells, probably by binding in cis to the Cμ1 domain of BCR-IgM in the steady-state through α2,3- or α2,6 linked sialic acids (3). To understand the organization of CD22 and its association with the BCR, Wasim et al. investigate the effects of mutations at the glycosylation sites of CD22 on BCR signaling and their results show that mutations of N-glycan sites attenuate CD22 phosphorylation and increase BCR signaling in response to antigenic stimulation. Irons and Lay show that ST6Gal-1, a sialyl-transferase that constructs the α2, 6-sialyl linkage on cell surface and extracellular glycans, is a pro-survival factor for transitional B Cells in mice.

B cell activation and differentiation is also critically dependent on signaling through pattern recognition receptors and chemokine receptors. Although BCR-mediated signaling pathways are largely distinct from Toll-like receptor (TLR) signaling pathways, these two pathways interact through shared signaling molecules, such as STAT3 activation by DOCK8-MyD88-Pyk2 complex (4). TLR-mediated activation even induces rapid reorganization of the IgM-BCR complex in B-1a cells (5). In this Research Topic, Rip et al. show that the high level of BTK expression in B cells enhances their sensitivity to TLR stimulation, suggesting that BTK promotes the synergistic activation of BCR and TLR engagements. In another direction, Maity et al. discusses how the cross-talks between chemokine receptor CXCR4 and the BCR of different immunoglobulin isotypes control the development and survival of leukemic B cells.

The BCR has also a close relationship with the actin cytoskeleton. BCR activation leads to reorganization of the cortical actin and conversely, changes in the actin cytoskeleton influence BCR signaling. Li et al. review the coordination between the actin cytoskeleton and BCR signaling, highlighting the potential role of actin in the initiation of BCR triggering. Actin remodeling is also important for the antigen extraction at the immunological synapse (IS) of B cells. Ibañez-Vega et al. show that proteasome activity is required for the dispersion of actin at the centrosome, which is important for lysosome recruitment to the IS, antigen extraction, and antigen presentation. Germinal center (GC) B cells extract antigen using multiple small peripheral BCR clusters instead of a single large IS seen in naïve or memory B cells. Actin remodeling is very critical for antigen extraction by GC B cells, relating actin-generated extraction forces to selection of high affinity GC B cells. The actin regulators during the GC response are reviewed by He and Westerberg.

Regulation of migration critically determines B cell development and functions. In this topic, Alsufyani et al. show the role of Mst1 and Mst2, mammalian orthologs of Hippo proteins, in B cell migration and homing. Follicular B cells lacking both Mst1 and Mst2 were shown to be unable to recirculate beyond spleen. It is also likely that Mst1 is required for migration of precursors of B-1a cells as B-1a cell development was defective in Mst1^−/−^ mice. Focusing on IgM deficiency in patients with novel mutations in *BTK* and *BLNK*, Geier et al. show that even hypomorphic mutations in these genes can lead to impaired B cell homeostasis.

With aging, bone marrow B cell production is inefficient and altered B cell homeostasis leads to expansion of activated B cells with atypical characteristics such as the expression of CD11b, CD11c, and T-bet. These B cells, termed as age-associated B cells (ABCs) and reviewed by Ma et al., accumulate with aging and show altered BCR repertoire. ABCs may play a significant role in autoimmune diseases by secreting autoreactive Abs and inducing Th17 cell differentiation. Ambegaonkar et al. shows that T-bet^+^ ABCs are induced by prolonged antigen stimulation in the presence of CpG and IFNγ.

We thank all the authors and reviewers for their invaluable work, which made the publication of this Research Topic possible. We hope that this collection of articles will serve as a useful source of knowledge to those interested in B cell activation and pathology.

## Author Contributions

TK made a substantial contribution when drafting this editorial essay. All authors listed have made direct intellectual contributions to the work, and approved it for its publication.

### Conflict of Interest

The authors declare that the research was conducted in the absence of any commercial or financial relationships that could be construed as a potential conflict of interest.
